# Early- and Late-Onset Preeclampsia: A Comprehensive Cohort Study of Laboratory and Clinical Findings according to the New ISHHP Criteria

**DOI:** 10.1155/2019/4108271

**Published:** 2019-09-17

**Authors:** Anna Wójtowicz, Małgorzata Zembala-Szczerba, Dorota Babczyk, Monika Kołodziejczyk-Pietruszka, Olga Lewaczyńska, Hubert Huras

**Affiliations:** ^1^Department of Obstetrics & Perinatology, Jagiellonian University Medical College, Ul. Kopernika 23, 31-501 Kraków, Poland; ^2^Department of Neonatology, Jagiellonian University Medical College, Ul. Kopernika 23, 31-501 Kraków, Poland

## Abstract

Recently, the diagnostic criteria of preeclampsia have been changed. No studies are available in the literature that analyzed in detail the differences between early-onset preeclampsia (EOP) and late-onset preeclampsia (LOP), taking into account the International Society for the Study of Hypertension in Pregnancy (ISSHP) criteria. Thus, we sought to retrospectively investigate in detail the differences in clinical and laboratory outcomes between EOP and LOP diagnosed according to the ISSHP criteria. A retrospective cohort study was conducted in 214 women with singleton pregnancies and preeclampsia admitted to the Department of Obstetrics and Perinatology of the University Hospital in Kraków, Poland, from 2013 to 2017 (113 (52.8%) women with EOP and 101 (47.2%) women with LOP). Electronic medical records were reviewed for demographics and medical history, laboratory tests, and delivery and neonatal data. Patients with preeclampsia accounted for 1.7% of the women who delivered during the study period. The EOP and LOP groups did not differ in the distribution of risk factors for preeclampsia. The most common risk factor was primiparity, which was observed in 72.0% of cases. Regarding the ISSHP diagnostic criteria, the two groups differed in the incidence of fetal growth restriction (*p*=0.0009), hemolysis (*p*=0.0416), and neurological complications (*p*=00342), which were found more often in the EOP group. In addition, the EOP group had more frequent occurrence of severe cardiorespiratory (*p* < 0.0001) and hematological (*p*=0.0127) complications, adverse fetoplacental conditions (*p* < 0.0001), and severe fetoplacental complications (*p*=0.0003). Children born to women with EOP had lower Apgar scores (*p* < 0.001) and higher rates of intraventricular hemorrhage (*p* < 0.0001), respiratory disorders requiring mechanical ventilation (*p* < 0.0001), and early (*p*=0.0004) and late sepsis (*p*=0.002). EOP differed from LOP in terms of maternal and perinatal adverse outcomes. The observed higher rates of fetoplacental adverse conditions and severe complications indicate a significant contribution of impaired placentation to the etiopathogenesis of EOP.

## 1. Introduction

Preeclampsia is a hypertensive disorder specific to pregnancy. Over the last decades, the incidence of preeclampsia has increased in some regions worldwide [1]. It complicates up to 5% of all pregnancies [2, 3] and is associated with serious maternal complications such as death, stroke, or liver rupture [4–6].

However, there has never been a consensus on the classification and diagnostic criteria for the hypertensive disorders of pregnancy. There are some differences between the two leading institutions dealing with the issue of hypertension in pregnancy, namely, American College of Obstetricians and Gynecologists (ACOG) and International Society for the Study of Hypertension in Pregnancy (ISSHP) [7–9], which can lead to differences in their observed rates of adverse maternal and fetal outcomes. In recent years, both ACOG and ISSHP have modified the diagnostic criteria for preeclampsia [7–9]. They have excluded the dependence of preeclampsia diagnosis on proteinuria. In 2013, ACOG published a report on hypertension in pregnancy, with fetal growth restriction (FGR) being eliminated from the consideration of preeclampsia [7]. In 2014, a revised statement from the ISSHP was published [8, 9]. In this statement, uteroplacental dysfunction manifesting as FGR is considered one of the preeclampsia diagnostic criteria. Furthermore, the end-organ dysfunction of preeclampsia, referred to as adverse conditions and severe complications, has been distinguished. Adverse conditions consist of maternal symptoms and abnormal laboratory and fetal monitoring results that may herald the development of severe maternal or fetal complications. In turn, severe maternal or fetal complications of preeclampsia are the features that warrant delivery. Depending on time, the condition is classified as early-onset preeclampsia (EOP), which requires delivery before 34 weeks' gestation, or late-onset preeclampsia (LOP), with delivery at or after 34 weeks or later [7–11].

Although the diagnostic criteria for EOP and LOP are the same, there are some uncertainties about the maternal and fetal outcomes [12, 13]. It is thought that EOP poses a high risk to both mother and fetus [14, 15], whereas LOP may present with less severe clinical symptoms [16]. Many studies have explored the clinical and laboratory findings in EOP and LOP. However, they mainly have focused on the risk factors and selected maternal and neonatal clinical outcomes as well as selected laboratory findings [17–26]. Moreover, previous studies utilized the diagnostic criteria of preeclampsia given several years ago.

Therefore, this study aimed to evaluate the differences in clinical and laboratory findings between patients with EOP and LOP and to assess whether both forms of the disease met the same ISSHP diagnostic criteria.

## 2. Materials and Methods

This retrospective cohort study included women with pregnancies and preeclampsia admitted to the Department of Obstetrics and Perinatology of the University Hospital in Kraków, Poland, from 2013 to 2017. Preeclampsia was diagnosed based on the International Society for the Study of Hypertension in Pregnancy (ISSHP) guidelines [8, 9]. The initial study population consisted of 231 patients with preeclampsia, accounting for 1.7% of the 13,716 patients who delivered at our institution from 2013 to 2017. EOP was diagnosed in 120 patients (52%), and 111 patients (48%) were diagnosed with LOP. Multiple pregnancies, which occurred at similar frequencies in the two groups (5.8% and 9.0%, respectively), were excluded from further analyses, and 113 women with EOP and 101 women with LOP were enrolled.

### 2.1. Management of Pregnancy Complicated by Preeclampsia

#### 2.1.1. Definitions

Gestational age was determined based on the date of the last menstrual period and/or the measurement of the crown-rump length in the first trimester of pregnancy.

Preeclampsia was diagnosed according to the criteria given in Table 1.

Diagnostic criteria for severe preeclampsia included the occurrence of severe uncontrolled hypertension (>160/110 mmHg) and any severe neurological, cardiorespiratory, hematological, renal, hepatic, or fetoplacental complications [8, 9]. Resistant preeclampsia was defined as the need for three antihypertensive medications for blood pressure control at ≥20 weeks of gestation [9].

HELLP was diagnosed if the platelet count is <10 × 10^9^/L, alanine aminotransaminase (ALT) or aspartate aminotransferase (AST) >70 IU/L, and lactate dehydrogenase (LDH) >600 IU/L [10].

In our center, all women with preeclampsia are referred to the hospital. When possible, on admission to the hospital with informed consent, a blood sample was collected to assess blood count, platelet count, and serum levels of creatinine, blood urea nitrogen, uric acid, and liver enzymes, and a urine sample was collected and analyzed for proteinuria. Depending on the clinical condition of the patient, 24-hour urine collection was performed if possible to assess the level of proteinuria. The number of women in whom specific measurements have been performed is given in the tables. Moreover, fetal well-being was evaluated through an ultrasound examination to determine the estimated fetal weight, Doppler flow in the umbilical artery (UA) and middle cerebral artery (MCA), cerebroplacental ratio (CPR), and nonstress cardiotocographic test (NST). We considered the pulsatility index (PI) in the UA and MCA as well as cerebroplacental ratio (CPR = MCA PI/UA PI).

Blood pressure was measured at least four times per day, and blood samples were collected 1-2 times per week. Fetal well-being was assessed based on fetal heart rate monitoring or NST. Ultrasound examination was performed at least once per week and in cases of Doppler abnormalities, every three days.

Patients were treated with the antihypertensive drug, including methyldopa as the first-line therapy. For emergency treatment of preeclampsia, labetalol and/or oral nifedipine were administered. Magnesium sulfate was administered for neuroprotection and prevention of seizures. Steroid therapy was given for lung maturation between 24 + 0 and 34 + 0 weeks of gestation.

Delivery was indicated in the event of preeclampsia after 37 weeks; placental abruption; progressive maternal renal, liver, neurological, or hematological dysfunction; inability to control maternal blood pressure despite antihypertensive medication; or nonreassuring cardiotocography or ultrasound-based concerns for fetal well-being or stillbirth.

The institutional review board waived the requirement for ethical approval for this analysis since the laboratory and sonographic evaluations were performed as an integral part of the routine clinical care, for which informed consent had been obtained from the women. Data were anonymized.

### 2.2. Statistical Analysis

Patient characteristics are described as means with standard deviation for normally distributed numerical data and as percentages for categorical variables. Differences were analyzed by Student's *t*-test for normally distributed data and the Mann–Whitney *U*-test for nonnormally distributed data. Chi-square and Fisher's exact tests were used for comparisons of categorical variables. In all analyses, *p* values <0.05 were considered statistically significant.

## 3. Results

The groups did not differ in terms of distribution of risk factors for preeclampsia (Table 2). The most common risk factor was primiparity, which was present in 72.0% of the patients. Considering the applied diagnostic criteria, the groups differed in the incidence of neurological complications (*p*=0.0342), hemolysis (*p*=0.0416), and FGR (*p*=0.0009) (Table 3).

On average, preeclampsia was diagnosed at week 30 in the EOP group and at week 36 in the LOP group. Admission-to-delivery interval was longer in the EOP group (8 ± 8.55 days) than in the LOP group (4 ± 5.5 days, *p*=0.0002); however, there was no difference in the delivery-to-discharge interval (Table 4).

Compared to the LOP group, the EOP group had a higher proportion of women with severe preeclampsia (96.4% vs. 87.0%, *p*=0.0412), higher mean systolic (178 mmHg vs. 168 mmHg, *p*=0.005) and diastolic blood pressure (109 mmHg vs. 104 mmHg, *p*=0.026) on admission, as well as resistant hypertension (30.0% vs. 2.0%; *p* < 0.0001), placental abruption (16.8% vs. 4.0%, *p*=0.004), diagnosis of genitourinary infection (27.4% vs. 15%, *p*=0.0385), and the need for albumin transfusion (19.4% vs. 8.0%, *p*=0.019) (Table 4). There were significant differences regarding the frequency of severe cardiorespiratory (*p* < 0.0001) and hematological complications (*p*=0.0127) (Table 5). There was one maternal death at 28 weeks of gestation because of pulmonary embolism as well as one case of hysterectomy due to placental abruption and uterine atony. Furthermore, complications in puerperium occurred more frequently in the EOP group than in the LOP group (56.0% vs. 41.6%, *p*=0.0375).

**Figure 1:**
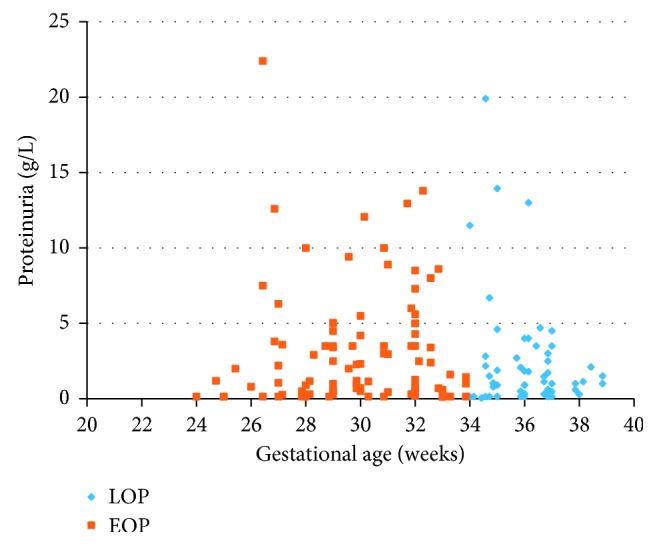
Proteinuria in women with early- and late-onset preeclampsia. EOP, early-onset preeclampsia; LOP, late-onset preeclampsia.

**Figure 2:**
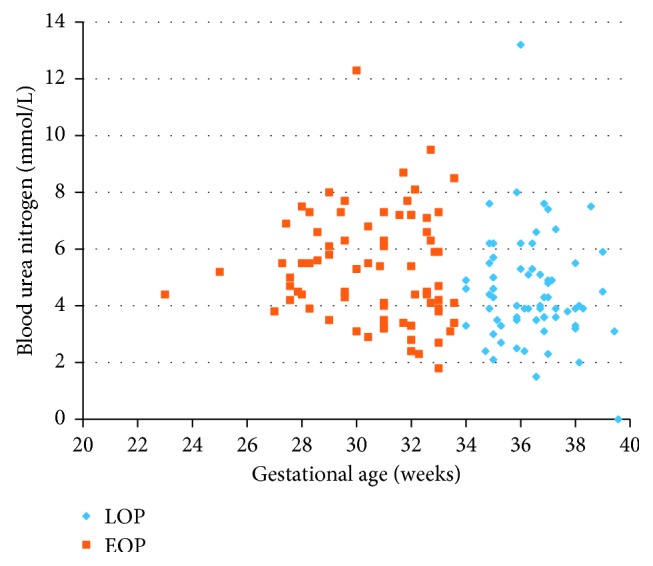
Blood urea nitrogen of women with early- and late-onset preeclampsia. EOP, early-onset preeclampsia; LOP, late-onset preeclampsia.

**Figure 3:**
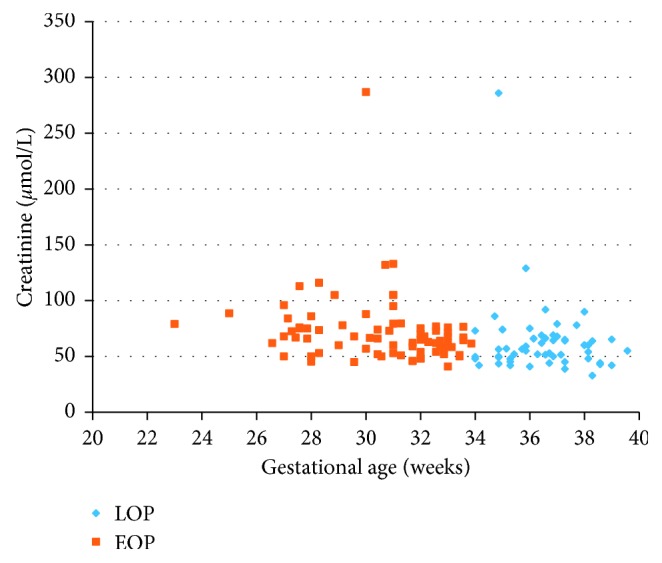
Creatinine of women with early- and late-onset preeclampsia. EOP, early-onset preeclampsia; LOP, late-onset preeclampsia.

All women had significant proteinuria, but patients in the EOP group were characterized by a significantly higher level of proteinuria (4.21 g vs. 2.32 g, *p*=0.007) (Figure 1), higher daily protein loss (6.35 g vs. 3.82 g, *p*=0.008), and more frequent daily protein loss ≥10 g (22.3% vs. 11.6%, *p*=0.0122) (Table 6). In addition, the EOP group demonstrated a higher blood urea nitrogen (5.31 vs. 4.88, *p*=0.021) (Figure 2) and serum creatinine concentration (72.3 vs. 63.0 IU, *p*=0.001) (Figure 3 and Table 6).

**Figure 4:**
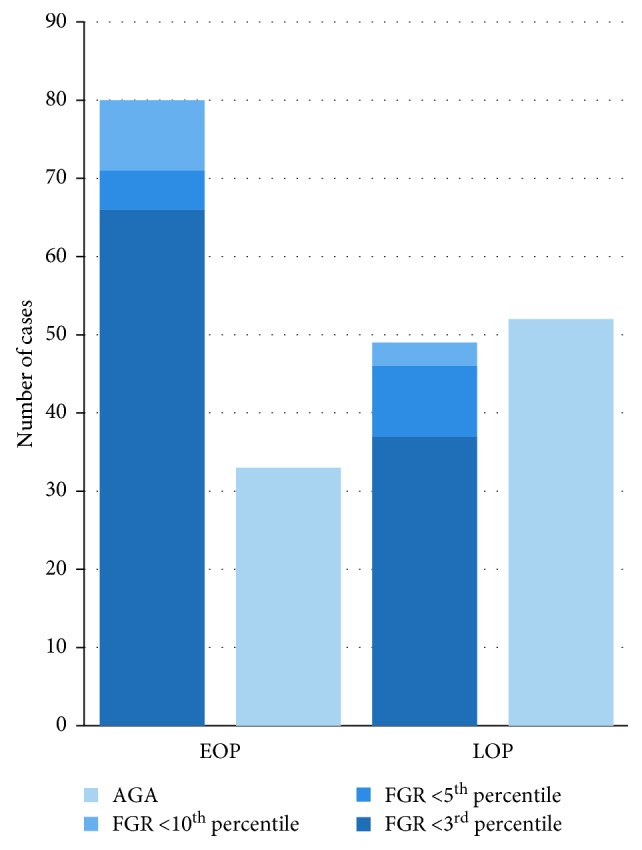
Distribution of fetal growth in women with early- and late-onset preeclampsia. AGA, appropriate for gestational age; EOP, early-onset preeclampsia; FGR, fetal growth restriction; LOP, late-onset preeclampsia.

The mean gestational age at birth and mean birth weight were significantly lower in the EOP group than in the LOP group (*p* < 0.001) (Table 7). The indication for delivery was intrauterine fetal distress in 69.0% of cases in the EOP group and in 33.0% of cases in the LOP group (*p* < 0.001) (Table 6). The study groups also differed in the prevalence of CPR below the 5^th^ percentile (70.0% vs. 32.0%, *p*=0.001) and abnormal MCA flow rate, defined as PI <5^th^ percentile (46.0% vs. 11.0%, *p* < 0.001). Moreover, compared to the LOP group, the EOP group had higher rates of FGR, defined as birth weight <10^th^ (*p*=0.001), 5^th^ (*p*=0.006), and 3^rd^ (*p*=0.002) percentiles, and lower Apgar scores in the 1^st^, 3^rd^, and 5^th^ minute (all *p* < 0.001) (Table 7 and Figure 4). The risk of birth of a child with an Apgar score <7/10 in the first minute instead of 10/10 was 7.59 times greater among patients with EOP than among patients with LOP (RR = 7.59, 95% CI = 3.11 – 18.53). Therefore, fetoplacental adverse conditions and severe complications were more frequent in the EOP group (Table 6).

Early preeclampsia was also associated with a higher risk of perinatal mortality (RR = 1.90, 95% CI: 1.20–3.01). In the EOP group, 22 (17.6%) women delivered at <28 weeks of gestation. There was one intrauterine death at 24 weeks of gestation, and 13 neonates (11.5%) died during the first month, of which 10 were born at ≤28 weeks and 3 after 28 weeks of gestation. All children born at <28 weeks of pregnancy developed respiratory distress syndrome and needed mechanical ventilation. Compared to the LOP group, the EOP group also had higher rates of intraventricular hemorrhage (27.4% vs. 1.0%, *p* < 0.0001), fresh frozen plasma transfusion (*p*=0.0002), and early (*p*=0.0004) and late sepsis (*p*=0.002) (Table 7).

## 4. Discussion

To the best of our knowledge, this is among the first studies that compared the clinical and laboratory outcomes between EOP and LOP, which were diagnosed according to the new ISHPP criteria. This comprehensive cohort study demonstrates that EOP and LOP do not meet the same diagnostic criteria. Early-onset preeclampsia poses a high risk of maternal neurological, cardiorespiratory, and hematological complications as well as adverse fetoplacental conditions and complications.

The incidence of preeclampsia in our department was 1.7%, which is consistent with the observation in the Chinese population [27] but lower than that reported in another report [17]. This relatively low prevalence of preeclampsia may be because our department is a tertiary referral center and admits mainly pregnant women at risk of giving birth before 32 weeks of gestation as well as the most severe cases, which accounted for 96.4% of patients in the EOP group and 87% of patients in the LOP group (*p*=0.0412). On the other hand, such a low percentage of pregnancies complicated by preeclampsia may result from ethnic conditions. It has been shown that there is a higher percentage of preeclampsia in the African American race than in the Chinese population [27]. The Polish population and our cohort are characterized by the fact that they are homogeneous of the Caucasian race. Another explanation may be that, in Poland, women are covered by medical care before the 10^th^ week of pregnancy, and medical appointments are held at least every 4 weeks. Another issue is the possibility of diversity in diet and vitamin supplementation.

The exact cause of preeclampsia is unknown, but maternal and placental factors are considered to be involved in the etiology of the disease. It has been suggested that EOP is more strongly associated with internal placental factors [28, 29], whereas the late-onset form may be primarily due to predisposing maternal factors. Although the present study did not find any differences between the two groups with respect to risk factors for preeclampsia, a previous study by Lisonkova and Joseph [17] found that older maternal age, unmarried status, and male sex of infant are typical to EOP and LOP, whereas African American race, chronic hypertension, and congenital anomalies are strongly associated with EOP [17]. Moreover, in the study by Aksornphusitaphong and Phupong [15], history of chronic hypertension was significantly associated with increased risk for only EOP, whereas a family history of chronic hypertension was associated with increased risk for only LOP. It is worth mentioning that patients in the studied population were predominantly primipara, which is known to be a major risk factor for preeclampsia [7].

It is thought that EOP may have a more severe course than LOP [8]. The incidence of placental abruption, overall mortality, and FGR have been shown to depend on the severity and duration of preeclampsia [30–34]. Our study did identify differences in the frequency of neurological complications and severe cardiorespiratory and hematological complications between the EOP and LOP groups. These findings are different from those of the studies by Pettit et al. [13] and Madazil et al. [19].

Interestingly, in our observation, the EOP group had higher serum levels of renal biomarkers than the LOP group, but no differences were found between the groups in terms of adverse renal conditions and severe complications. However, renal function can be impaired throughout preeclampsia as a result of glomerular endotheliosis, leading to a decrease in the glomerular filtration rate [35]. In the present study, the groups differed in terms of proteinuria, blood urea nitrogen, and serum creatinine levels, which are representative of renal function in pregnancy. In contrast to our study, Weitzner et al. [25] did not show differences in creatinine levels. Furthermore, the increased serum level of uric acid has been shown to correlate with the severity of glomerular endotheliosis [36]. However, in our study, the groups did not differ in terms of uric acid concentration, in contrast to a previous report by Li et al. comparing EOP and LOP in patients with severe hypertension [18].

In our observation, EOP was strongly associated with adverse fetoplacental conditions and severe complications. The cause of impaired fetoplacental function may be the abnormal invasion of trophoblasts and remodeling of the spiral arteries, which can result in limited blood flow and lead to growth restriction and fetal distress symptoms. In our study, FGR occurred in 60.2% of all pregnancies complicated by preeclampsia, which is consistent with the study of Madazil et al. [19], which reported a rate of 59.1%. Furthermore, in the present study, the incidence of fetal growth restriction reached as much as 70.7% in pregnancies with EOP. This finding is consistent with the report of Lisonkova and Joseph [17], but not with other observations [18]. Interestingly, the studied groups did not differ in terms of the frequency of flow disturbances in the UA, but in the EOP group, there were more frequent flow disturbances in the MCA. CPR <5^th^ percentile also occurred more frequently in the EOP group. Both our study and Sibai's study [30] showed a 100% mortality rate in children born before 28 weeks of pregnancy.

Compared to other papers, the major strength of the present work is that it contains a detailed, extensive analysis of clinical and laboratory factors that could be collected from the medical records. However, our study also has several limitations. First, it is a retrospective observational study with a relatively small sample size. Moreover, in some cases, due to the severity of the disease, it became necessary to terminate the pregnancy within a short period of time, which did not permit further laboratory testing. Finally, the hospital where the study was conducted is a tertiary referral center, wherein the most severe cases from the region of south-eastern Poland are treated; hence, it could affect the results.

In summary, EOP differed from LOP mainly in terms of adverse maternal and fetoplacental conditions and severe complications. The observed higher rates of FGR and vascular flow disturbances indicate a significant contribution of impaired placentation to the etiopathogenesis of the early form of preeclampsia. The obtained results indicate that we should pay attention to the neurological, cardiorespiratory, and hematological parameters as well as to the intensive fetal surveillance in women with EOP.

## Figures and Tables

**Table 1:** Diagnostic criteria for preeclampsia [8].

Blood pressure	Systolic blood pressure ≥140 mmHg and/or diastolic blood pressure ≥90 mmHg that are noted twice within 6 hours after 20 weeks of gestation in women with normal blood pressure before conception or in women with previous chronic hypertensive disorders

And coexistence of one or more of the following new-onset conditions	
Proteinuria	Spot urine protein/creatinine >30 mg/mmol (0.3 mg/mg) or >300 mg/day or at least 1 g/L (“2+”) on dipstick testing

Other maternal organ dysfunctions	(1) Renal insufficiency (creatinine >90 *μ*mol/L; 1.02 mg/dL)
	(2) Liver involvement (doubling of serum transaminases and/or severe right upper quadrant pain)
	(3) Neurological complications (eclampsia, altered mental status, blindness, stroke, or more commonly hyperreflexia when accompanied by clonus and severe headaches when accompanied by hyperreflexia and persistent visual scotomata)
	(4) Hematological complications (platelet count <150,000/dL, DIC, and hemolysis)

Uteroplacental dysfunction	Fetal growth restriction

DIC, disseminated intravascular coagulation.

**Table 2:** The distribution of selected maternal risk factors for preeclampsia in women with singleton pregnancy and early- or late-onset preeclampsia.

Risk factors for preeclampsia	EOP (*n* = 113)	LOP (*n* = 101)	Total (*n* = 214)
Primiparity, *n* (%)	83 (73.4)	71 (70.3)	154 (72.0)
Multiparity (>3), *n* (%)	3 (2.6)	5 (5.0)	8 (3.7)
Previous preeclamptic pregnancy, *n* (%)	2 (1.7)	2 (2.0)	4 (1.8)
Chronic hypertension, *n* (%)	20 (17.7)	14 (14.0)	34 (15.8)
Chronic renal disease, *n* (%)	4 (3.5)	2 (2.0)	6 (2.8)
History of thrombophilia, *n* (%)	2 (1.7)	1 (1.0)	3 (1.4)
In vitro fertilization, *n* (%)	2 (1.7)	3 (3.0)	5 (2.3)
Family history of preeclampsia, *n* (%)	Data not available	Data not available	Data not available
Type 1 or type 2 diabetes mellitus, *n* (%)	7 (6.2)	8 (8.0)	15 (7.0)
Obesity, BMI >30 kg/m^2^, *n* (%)	13 (11.5)	12 (12.0)	25 (11.7)
Systemic lupus erythematosus, *n* (%)	1 (0.9)	0 (0.0)	1 (0.4)
Maternal age ≥40 years, *n* (%)	9 (8.0)	4 (4.0)	13 (6.0)

BMI, body mass index; EOP, early-onset preeclampsia; LOP, late-onset preeclampsia.

**Table 3:** The revised ISSHP criteria of preeclampsia [8, 9] in women with singleton pregnancy and early- or late-onset preeclampsia.

Criterion	EOP (*n* = 113)	LOP (*n* = 101)	Total (*n* = 214)	*p*
Proteinuria	113 (100.0)	101 (100.0)	214 (100.0)	ns
Renal insufficiency (creatinine >90 *μ*mol/L), *n* (%)	11 (9.7)	6 (6.0)	17 (7.9)	ns
Liver involvement, *n* (%)	15 (13.3)	8 (7.9)	23 (10.7)	ns
Neurological complications, *n* (%)	20 (17.7)	8 (8.0)	28 (13.0)	0.0342
Hematological complications, *n* (%)	50 (44.2)	35 (34.6)	85 (39.7)	ns
(i) Thrombocytopenia, *n* (%)	37 (74)	29 (82.8)	66 (77.6)	ns
(ii) DIC, *n* (%)	0 (0.0)	2 (5.8)	2 (2.4)	ns
(iii) Hemolysis, *n* (%)	13 (26)	4 (11.4)	17 (20.0)	0.0416
FGR, *n* (%)	80 (70.7)	49 (48.5)	129 (60.3)	0.0009

DIC, disseminated intravascular coagulation; EOP, early-onset preeclampsia; FGR, fetal growth restriction; ISSHP, International Society for the Study of Hypertension in Pregnancy; LOP, late-onset preeclampsia; ns, nonstatistically significant.

**Table 4:** Characteristics and occurrence of adverse maternal outcomes in women with early-onset and late-onset preeclampsia.

	EOP (*n* = 113)	LOP (*n* = 101)	Total (*n* = 214)	*p*
Maternal age at EDD, years ± SD (range)	30.7 ± 5.5 (19.00–48.00)	30.1 ± 5.3 (19.00–47.00)	30.43 ± 5.4 (19.00–48.00)	ns
Gestational age at inclusion, weeks ± SD (range)	30.0 ± 2.5 (22.0–33.0)	36.2 ± 1.4 (34.0–39.3)	33.1 ± 1.6 (22.0–39.3)	0.00001
Systolic blood pressure on admission, mmHg ± SD (range)	178 ± 18 (140–240)	168 ± 18 (120–230)	173 ± 18 (120–240)	0.005
Diastolic blood pressure on admission, mmHg ± SD (range)	109 ± 12 (90–150)	104 ± 11 (70–145)	106 ± 11 (70–150)	0.026
Antihypertensive drug administration, *n* (%)				
Methyldopa	106 (93.8)	90 (90.0)	196 (94.8)	ns
Calcium channel blocker	46 (40.7)	29 (29.0)	75 (35.0)	ns
Beta-blocker	65 (57.5)	26 (26.0)	91 (42.5)	<0.001
Resistant hypertension, *n* (%)	34 (30.0)	2 (2.0)	36 (16.8)	<0.0001
MgSO_4_ administered, *n* (%)	65 (57.5)	30 (30.0)	85 (45.8)	<0.001
Admission-to-delivery interval, days ± SD (range)	6.8 ± 6.8 (1–30)	6 ± 8.5 (1–53)	6 ± 7.0 (1–53)	0.021
Gestational age at delivery, weeks ± SD (range)	30.6 ± 2.2 (23.0–33.8)	36.6 ± 1.4 (34.0–39.5)	33.9 ± 1.9 (23.0–39.5)	<0.0001
Delivery-to-discharge interval, days ± SD (range)	6.7 ± 3.5 (3–24)	6.4 ± 3.6 (3–30)	6.5 ± 3 (3–30)	ns
Severe preeclampsia, *n* (%)	109 (96.4)	87 (87.0)	196 (91.5)	0.0412
HELLP, *n* (%)	5 (4.4)	4 (4.0)	9 (4.2)	ns
Eclampsia before delivery, *n* (%)	0 (0.0)	2 (2.0)	2 (0.9)	ns
Postpartum eclampsia, *n* (%)	17 (15.0)	7 (7.0)	24 (11.2)	ns
Placental abruption, *n* (%)	19 (16.8)	4 (4.0)	23 (10.7)	0.004
Hemorrhage, *n* (%)	3 (2.6%)	1 (1.0)	4 (1.8)	ns
Blood transfusion, *n* (%)	12 (10.6)	7 (7.0)	19 (8.8)	ns
Albumin transfusion, *n* (%)	22 (19.4)	8 (8.0)	30 (14.0)	0.019
Anemia, *n* (%)	50 (44.2)	40 (40.0)	90 (42.0)	ns
Pulmonary edema, *n* (%)	5 (4.4)	0 (0.0)	5 (2.3)	ns
Hysterectomy, *n* (%)	0 (0.0)	1 (1.0)	1 (0.4)	ns
Maternal death, *n* (%)	1 (0.8)	0	1 (0.4)	ns
DIC, *n* (%)	0 (0.0)	2 (2.0)	2 (0.9)	ns
ICU, *n* (%)	22 (19.4)	6 (6.0)	28 (13.0)	0.02
Uterine contraction disorders, *n* (%)	3 (2.6)	1 (1.0)	4 (1.8)	ns
Thrombosis, *n* (%)	2 (1.7)	4 (4.0)	6 (2.8)	ns
Healing disorders of the scar, *n* (%)	3 (2.6)	6 (6.0)	9 (4.2)	ns
Genitourinary infection, *n* (%)	31 (27.4)	15 (15.0)	46 (21.5)	0.0385

DIC, disseminated intravascular coagulation; EDD, estimated date of delivery; EOP, early-onset preeclampsia; HELLP, hemolysis, elevated liver enzymes, low platelets; ICU, intensive care unit; LOP, late-onset preeclampsia; ns, nonstatistically significant; SD, standard deviation.

**Table 5:** Adverse conditions and severe complications in women with early-onset and late-onset preeclampsia.

Organ system affected	EOP (*n* = 113)		LOP (*n* = 101)		*p*	
	Adverse conditions	Severe complications	Adverse conditions	Severe complications	Adverse conditions	Severe complications
CNS, *n* (%)	3 (2.6)	17 (15.0)	1 (1.0)	7 (7.0)	ns	ns
Cardiorespiratory, *n* (%)	2 (1.7)	34 (30.0)	1 (1.0)	2 (2.0)	ns	<0.0001
Hematological, *n* (%)	37 (32.7)	24 (21.2)	29 (29.0)	9 (9.0)	ns	0.0127
Renal, *n* (%)	15 (13.2)	2 (1.7)	8 (8.0)	1 (1.0)	ns	ns
Hepatic, *n* (%)	15 (13.2)	0 (0.0)	8 (8.0)	0 (0.0)	ns	ns
Fetoplacental, *n* (%)	87 (77.0)	21 (18.5)	42 (42.0)	3 (3.0)	<0.0001	0.0003

CNS, central nervous system; EOP, early-onset preeclampsia; LOP, late-onset preeclampsia; ns, nonstatistically significant.

**Table 6:** Laboratory measurements in women with early-onset and late-onset preeclampsia.

	EOP (*n*)^*∗*^, mean ± SD	LOP (*n*)^*∗*^, mean ± SD	*p*
Proteinuria (g/L)	(*n* = 80) 4.21 ± 6.84	(*n* = 58) 2.32 ± 3.61	0.007
Proteinuria, *n* (%)	(91)	(78)	
≤5 g/day, *n* (%)	53 (58.2)	59 (75.6)	ns
5.1–9.9 g/day, *n* (%)	16 (17.6)	10 (12.8)	ns
≥10 g/day, *n* (%)	22 (24.2)	9 (11.6)	0.0122
24-hour urine proteinuria (g/24 h)	(*n* = 91) 6.35 ± 8.23	(*n* = 78) 3.82 ± 4.36	0.008
Blood urea nitrogen (mmol/L)	(*n* = 75) 5.31 ± 1.93	(*n* = 62) 4.88 ± 3.14	0.021
Uric acid (*µ*mol/L)	(*n* = 30) 418.1 ± 107.6	(*n* = 25) 389.6 ± 82.3	ns
Creatinine (*µ*mol/L)	(*n* = 78) 72.3 ± 31.2	(*n* = 56) 63.0 ± 34.4	0.001
Creatinine clearance (ml/min)	(*n* = 16) 102.8 ± 48.0	(*n* = 15) 127.8 ± 74.1	ns
Total serum protein (g/L)	(*n* = 71) 55.8 ± 6.0	(*n* = 48) 57.7 ± 7.21	ns
Albumin (g/L)	(*n* = 50) 29.2 ± 4.28	(*n* = 30) 31.5 ± 6.74	ns
Hemoglobin (g/L)	(*n* = 113) 12.3 ± 1.49	(*n* = 92) 12.3 ± 1.33	ns
Hematocrit (%)	(*n* = 113) 35.6 ± 4.3	(*n* = 92) 35.8 ± 3.7	ns
Erythrocytes (×10^9^/L)	(*n* = 113) 4.05 ± 0.47	(*n* = 92) 4.04 ± 0.42	ns
Platelet (×10^9^/L)	(*n* = 113) 184.0 ± 72.8	(*n* = 92) 178.0 ± 68.9	ns
Alanine aminotransaminase (U/L)	(*n* = 73) 43.4 ± 48.2	(*n* = 50) 70.9 ± 141.0	ns
Aspartate aminotransferase (U/L)	(*n* = 73) 44.8 ± 53.3	(*n* = 50) 65.2 ± 134.9	ns

The values in ( )^*∗*^ indicate number of performed measurements; EOP, early-onset preeclampsia; LOP, late-onset preeclampsia; ns, nonstatistically significant; SD, standard deviation.

**Table 7:** Fetal factors associated with early- and late-onset preeclampsia.

	EOP (*n* = 113)	LOP (*n* = 101)	Total (*n* = 214)	*p*
Gestational age at delivery, weeks ± SD (range)	30.6 ± 2.2 (23.0–33.8)	36.6 ± 1.4 (34.0–39.5)	33.9 ± 1.9 (23.0–39.5)	<0.0001
Birth weight (g), mean ± SD (range)	1358 ± 497 (460–3450)	2511 ± 689 (1010–4320)	1934 ± 592 (460–4320)	<0.001
Fetal sex				
Female, *n* (%)	63 (56.0)	53 (52.0)	116 (54.0)	ns
Male, *n* (%)	50 (44.0)	48 (48.0)	98 (46.0)	ns
Intrauterine fetal distress, *n* (%)	78 (69.0)	33 (33.0)	111 (51.8)	<0.001
UA PI >95^th^ percentile, *n* (%)	27 (23.9)	15 (15.0)	42 (19.6)	ns
MCA PI <5^th^ percentile, *n* (%)	52 (46.0)	11 (11.0)	63 (29.4)	<0.001
CPR <5^th^ percentile, *n* (%)	79 (70.0)	32 (32.0)	111 (51.8)	0.001
FGR <10^th^ percentile, *n* (%)	80 (70.7)	49 (49.0)	129 (60.2)	0.0015
FGR <5^th^ percentile, *n* (%)	71 (62.8)	46 (46.0)	117 (54.6)	0.006
FGR <3^rd^ percentile, *n* (%)	66 (58.4)	37 (37.0)	103 (48.1)	0.002
Apgar score at 1 min, mean ± SD (range)	6.7 ± 1.9 (1–10)	9.1 ± 1.4 (1–10)	7.9 ± 1.5 (1–10)	<0.001
Apgar score at 3 min, mean ± SD (range)	7.4 ± 1.7 (1–10)	9.6 ± 0.7 (7–10)	8.5 ± 1.4 (1–10)	<0.001
Apgar score at 5 min, mean ± SD (range)	7.8 ± 1.3 (4–10)	9.8 ± 0.6 (7–10)	8.8 ± 1.3 (4–10)	<0.001
Apgar score <7 at 1 min, *n* (%)	51 (45.0)	4 (4.0)	55 (25.7)	<0.001
Apgar score <7 at 3 min, *n* (%)	27 (23.9)	2 (2.0)	29 (13.5)	<0.001
Apgar score <7 at 5 min, *n* (%)	19 (16.8)	0 (0.0)	19 (8.8)	<0.001
Intrauterine fetal death, *n* (%)	1 (0.9)	0 (0.0)	1 (0.4)	ns
IVH, *n* (%)	31 (27.4)	1 (1.0)	32 (14.9)	<0.0001
FFP transfusion, *n* (%)	15 (13.3)	0 (0.0)	15 (7.0)	0.0002
Mechanical ventilation	33 (29.2)	1 (1.0)	34 (15.9)	<0.0001
Infection complications, *n* (%)				
Early sepsis	18 (16.0)	1 (1.0)	19 (8.8)	0.0004
Late sepsis	20 (17.7)	2 (2.0)	22 (10.2)	0.0021
Retinopathy, *n* (%)	2 (1.8)	0 (0.0)	2 (0.9)	ns
NEC, *n* (%)	3 (2.6)	0 (0.0)	3 (1.4)	ns
Fetal death, *n* (%)	13 (11.5)	0 (0.0)	13 (6.0)	0.0008
Born ≤28 weeks	10 (8.8)	0 (0.0)		0.0058
Born >28 weeks	3 (2.6)	0 (0.0)		ns

CPR, cerebroplacental ratio; FFP, fresh frozen plasma; FGR, fetal growth restriction; IVH, intraventricular hemorrhage; MCA, middle cerebral artery; NEC, necrotizing enterocolitis; ns, nonstatistically significant; PI, pulsatility index; RDS, respiratory distress syndrome; SD, standard deviation; UA, umbilical artery; EOP, early-onset preeclampsia; LOP, late-onset preeclampsia.

## Data Availability

Medical data are archived on the platform of the University Hospital (https://www.su.krakow.pl).
